# Laboratory animal welfare and human attitudes: A cross-sectional survey on heterospecific play or “rat tickling”

**DOI:** 10.1371/journal.pone.0220580

**Published:** 2019-08-14

**Authors:** Megan R. LaFollette, Sylvie Cloutier, Colleen Brady, Brianna N. Gaskill, Marguerite E. O’Haire

**Affiliations:** 1 Department of Animal Sciences, College of Agriculture, Purdue University, West Lafayette, Indiana, United States of America; 2 Independent Researcher, Ottawa, Ontario, Canada; 3 Department of Agricultural Sciences Education and Communication, College of Agriculture, Purdue University, West Lafayette, Indiana, United States of America; 4 Center for the Human-Animal Bond, Department of Comparative Pathobiology, College of Veterinary Medicine, Purdue University, West Lafayette, Indiana, United States of America; Universidade do Porto Instituto de Biologia Molecular e Celular, PORTUGAL

## Abstract

**Introduction:**

Laboratory rat welfare is critically influenced by laboratory animal personnel through their implementation, or lack of implementation, of various enrichment techniques. One such promising technique is heterospecific play, or “rat tickling”, which mimics aspects of rat rough-and-tumble play and can contribute to improving welfare, but may be infrequently implemented. The theory of planned behavior can be used to study implementation by measuring intentions and beliefs about rat tickling, including behavioral attitudes (whether it is good or bad), subjective norms (whether there is social/professional pressure to provide it), and control beliefs (whether they feel in control of providing it). Therefore, the objective of this study was to identify current rat tickling prevalence and predictors among laboratory animal personnel in the United States and Canada. Our hypothesis was that rat tickling prevalence would be low and associated with beliefs about the practice, enrichment, and laboratory animals in general.

**Methods:**

Laboratory animal personnel were recruited from widespread online promotion. A total of 794 personnel (mean = 40±11 years, 80% white, 80% female) completed at least 50% of the mixed methods online survey and met inclusion criteria of currently working with laboratory rats in the USA or Canada. The survey included questions about demographics, enrichment practices and beliefs, attitudes towards rats, general positive behaviors (e.g. talking to laboratory animals), and both practices and beliefs about rat tickling. Qualitative data were coded using thematic analysis. Quantitative data were analyzed using general linear models.

**Results:**

Laboratory personnel reported low levels of rat tickling implementation, with 89% of participants reporting using it never or rarely. Laboratory personnel reported 2 key benefits (handling: 61%, welfare: 55%) and 3 key barriers (time: 59%, personnel: 22%, and research: 22%) to rat tickling using qualitative analysis. Current and planned rat tickling were positively associated with more positive beliefs (social/professional pressure p<0.0001, control of providing tickling p<0.0001) and familiarity with tickling (p<0.0001). Current rat tickling was also positively associated with more positive general behaviors towards laboratory animals, such as naming animals (p<0.0001). Future rat tickling was positively associated with more positive attitudes about it (p<0.0001) and a desire to implement more enrichment (p<0.01).

**Conclusion:**

Our findings show that even though rat tickling implementation is currently low, it is positively associated with personnel beliefs, familiarity, general attitudes, and a desire for more enrichment. That is, laboratory animal personnel were more likely to provide rat tickling if they were more familiar with it, thought providing it was both good and under their control, and felt subject to social/professional pressure, as well as if they wanted to provide more enrichment and generally had more positive behaviors towards laboratory animals. There is potential to increase rat tickling by increasing personnel familiarity with the procedure through training, decreasing the time required, and changing personnel beliefs–thereby improving rat welfare.

## Introduction

To facilitate various types of basic, applied, and regulatory research, a number of animals are housed in laboratories. In this captive setting, these laboratory animals may experience stress as a result of housing, husbandry, and research practices [1]. To mitigate these stressors it is recommended that captivate animals receive biologically relevant enrichments and handling improvements[2]. Laboratory animal personnel are often the individuals responsible for implementing or recommending these enrichments and thereby improving animal well-being through their direct or indirect actions. These actions may either be supported or hindered as a result of many factors, which may include personnel role (e.g. animal care technician, laboratory manager, clinical veterinarian, principal investigator), institution type (e.g. universities, contract research organizations, government research agencies), or specific research type.

Handling of laboratory animal during everyday care or research protocols is often an underestimated source or stress that can cause unintended variability in data within and between laboratories and affect animal welfare. Handling can result in increased heart rate, corticosterone levels, glucose, and more [3]. Rats, one of the most common laboratory animals, experience stress from handling [4,5] which can make handling difficult and contribute to poor animal welfare. Fortunately, handling can be improved by implementing habituation techniques such as heterospecific play or “rat tickling.” Rat tickling is a human-animal interaction that mimics aspects of rat rough-and-tumble play [6]. It is more effective than exposure to a passive hand or minimal handling [7] and more efficient than other habituation techniques [8]. It increases rat positive affect, habituation, and positive approach behaviors thus reducing routine handling stress [7].

Despite the known benefits of rat tickling, its current level of implementation and barriers to more widespread implementation are unknown [7]. However, it is suspected that the prevalence of rat tickling implementation is relatively low, which would indicate that many rats are not receiving an enrichment that could be beneficial to their welfare. Anecdotally, laboratory animal personnel state that several factors prevent its widespread use (including its perceived time intensive nature, disbelief or lack of knowledge in its beneficial effects, and even the name “rat tickling” itself), but there is no scientific evaluation of these statements [7]. Regardless of their specific reasons, rat tickling provision is ultimately a behavioral decision made by laboratory animal personnel.

Scientifically evaluating, understanding, and predicting human behavior can be complex and challenging. Fortunately, the theory of planned behavior has been successfully used across a wide variety of target behaviors [9]. This theory is the explicit basis for over 832 published studies, is highly predictive, and can be used to develop interventions for behavior change in humans [9]. The basis of this theory is that humans are more likely to perform behaviors when they plan to do them. In turn, those plans (or intentions) to behave in a certain way can be predicted using three main factors: beliefs about the consequences of a behavior (behavioral attitudes); beliefs about social and professional pressures to perform the behavior (subjective norms); and beliefs about control over performing the behavior (perceived behavioral control) [10]. By measuring these factors, researchers can determine which beliefs may be the best targets for interventions aiming at increasing the performance of a behavior.

In the field of animal welfare, the theory of planned behavior has been used to evaluate the impact of stockperson beliefs on farm animal welfare, develop an intervention to change these beliefs, and, in turn, improve farm animal welfare [11]. Stockpeople with more negative beliefs about farm animals and animal handling were more likely to handle them roughly which led to decreased animal productivity and welfare [12]. However, when stockpeople were re-trained using an intervention focusing on improving their beliefs about the animals and their handling–based on areas identified using the theory of planned behavior–they actually changed their behavior which, in turn, improved both farm animal productivity and welfare [13].

Our objective in this study was to characterize the current use of and beliefs about rat tickling in the status quo. Our specific aims were to (a) quantify the current prevalence of rat tickling and how it is used and (b) identify predictors that influence both intentions and past frequency of providing rat tickling including using the theory of planned behavior. Based on previous research using the theory of planned behavior and personal experience, we hypothesized that rat tickling will be provided more frequently by laboratory personnel with more positive attitudes towards rats and general behaviors towards laboratory animals, more familiarity, and more positive beliefs about rat tickling. With this knowledge, we hope to identify promising areas for future research and interventions to increase rat tickling prevalence therby improving rat welfare.

## Materials and methods

All procedures and informed consent protocols were approved by Purdue University’s Human Research Protection Program Institutional Review Board, protocol #1712020004. No interactions occurred between the research team and animals during the course of the study; therefore, we did not seek approval from Purdue University’s Institutional Animal Care and use Committee (IACUC).

### Participants & procedures

Participants were recruited between February 22^nd^ and March 26^th^, 2018 via widespread online promotions designed to maximize sample size [14]. Online contacts were through seven modalities: direct emails to known laboratory personnel, list serves (e.g., CompMed, LAREF, etc.), email lists (e.g., CALAS, MSMR), Facebook groups/pages/personal accounts (e.g. Laboratory Animal Sciences, Dog Spies), LinkedIn groups/personal pages (e.g., AALAS, Animal Behavioral Biology), website advertising (CALAS & AALAS,) and online webinars (e.g., AALAS). All modalities were contacted up to four times with the same study flyer following recommended survey procedures [15]. Additionally, all materials were translated into French by a native French Canadian. Following voluntary informed consent, participants completed a 30 min online survey. For compensation for their time, participants could be entered into a drawing for a choice between $40 Amazon gift card or cash (chosen by 62.5% and 37.5%, respectively). Participants were included if they were over the age of 18 and report current work with laboratory rats in the United States or Canada.

### Measures

This survey was developed by reviewing literature and consulting with experts in survey methodology, behavior theory, and laboratory animal enrichment. When possible, validated instruments were used (i.e. theory of planned behavior survey). When validated instrumentation did not exist, previous work was modified or new items were created, reviewed by experts, piloted, and revised as necessary. The survey question text and scales are available in **S1 Table**.

#### Demographics & work factors

Participants were asked about their demographics, current work, and percentage of time spent working with rats. Demographics included age, gender, race, and highest level of education. Current work questions related to current country of work (i.e. United States or Canada), role (e.g., animal care technician, veterinarian), type of institution (e.g., academic, contract research organization), primary type of research (e.g. applied, basic, regulatory), and both years and hours per week working with laboratory animals. Participants were informed that work was defined broadly and may include hands-on work such as changing cages or running procedures or hands-off work such as running a laboratory or research studies.

#### Enrichment, attitudes, & general behaviors

Enrichment use was evaluated with questions about general enrichment factors and frequency of using rat tickling. For general enrichment factors, participants were asked their degree of control over enrichment and if they wished they could provide more enrichment than they currently provided. At the beginning of this survey section, to counter the possibility of participants having different definitions or misunderstandings of enrichment, participants were instructed that “in this study, we consider animal enrichment to be any attempt to improve animal welfare by enhancing the quality of a captive animal’s care by providing stimuli necessary for psychological and physical well-being” [16].

Participants also received a Rattitude survey to assess their general attitudes towards rats and a general behavior survey, both adapted from Hemsworth & Coleman [11]. Participants were asked if they agreed or disagreed with statements about laboratory rats, five negative (e.g rats are smelly) and five positive (e.g. rats are entertaining). For general behaviors, participants were asked if they agreed or disagreed that they often observe, pet, talk to, or name their laboratory animals.

#### Rat tickling information

**Fig 1:**
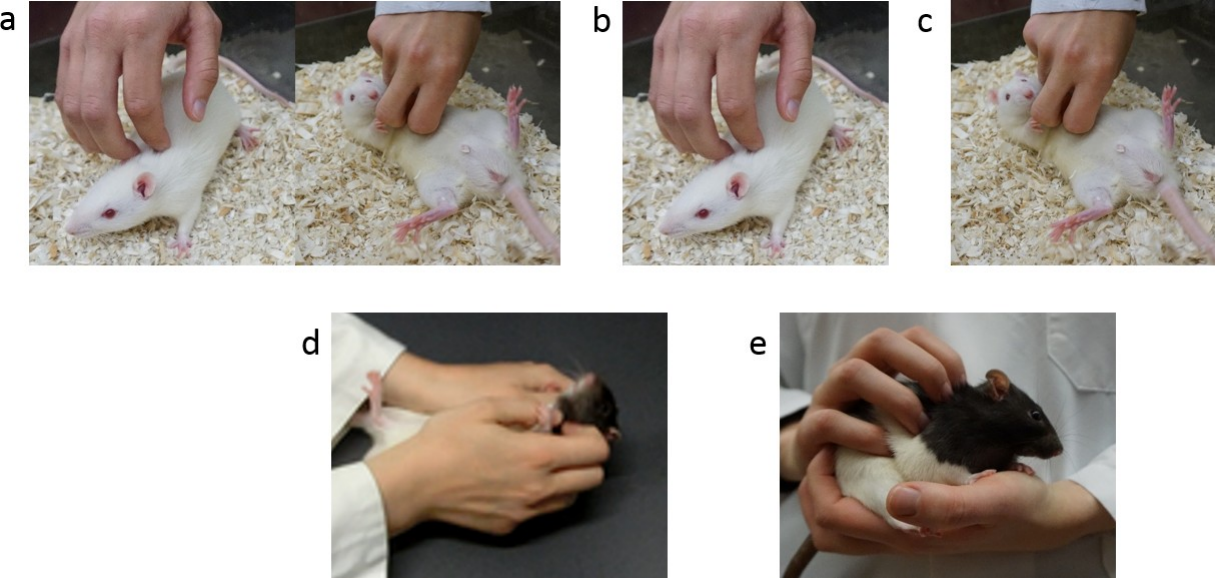
Pictorial tickling procedures. A: Dorsal contact and pin (standard, validated rat tickling procedure), B: Dorsal contact only or stroking in the cage, C: Pin only, D: Two-handed pin only, E: Stroking in the hand.

Current knowledge and use of rat tickling were evaluated via questions about current frequency of provision and familiarity with rat tickling. If participants were at least a little familiar with the technique, they were asked to select a pictorial representation of the technique used in their lab (**Fig 1**). The final question was included because anecdotally some individuals say they use rat tickling, but describe techniques that do not mimic aspects of rat social play, which is the basis of effective rat tickling.

At the end of this survey section, to counter the possibility of participants having misunderstandings or no knowledge of rat tickling–and prepare participants for the theory of planned behavior section–participants were instructed that “in this survey, rat tickling is defined as an interaction between a human and rat that mimics aspects of rat social play.”

#### Beliefs: Theory of planned behavior

The theory of planned behavior was used to assess rat tickling intentions, behavioral attitudes, subjective norms, and perceived behavioral control. Surveys constructed using this theory typically have excellent reliability and validity [9].

First, participants were asked open-ended, qualitative questions to allow participants to reply with their most salient answers without additional prompting. These questions were modeled after methods used in an elicitation study for the theory of planned behavior [9] and included asking participants what makes it difficult to tickle rats, easy to tickle rats, and what are the advantages to rat tickling, if any.

Then, participants were asked close-ended, quantitative questions in 10 sections that directly (and indirectly) assessed behavioral intentions, attitudes (behavioral beliefs x outcome evaluations), subjective norms (normative beliefs x motivation to comply), and perceived behavioral control (control belief power x control belief strength). Information on all items (including mean ± standard deviation, scale, summary scores, and Cronbach’s alpha) is included in **S5 Table** and **S6 Table**. This survey was developed using a manual for constructing questionnaires based on the theory of planned behavior [9]. At least 3 items were measured for each construct. Summary variables were calculated using theory of planned behavior protocols [9]. Overall, only one item was dropped from the survey based on its extremely low reliability, which was likely due to it being the only item that was negatively worded in the series (i.e., The decision to provide rat tickling to laboratory rats is… “*beyond my control*” versus “completely up to me” or “I am confident I can provide rat tickling”.)

### Data analysis

#### Variable coding

To ensure that all descriptive data reporting and summary scores indicate the same responses, only participants that answered 50% of questions in the survey were included for analysis. When comparing these participants to all respondents who started the survey, no obvious visual differences in demographics were seen.

Categorical data options that contained less than 20 responses were collapsed into larger categories to assist with analysis. Similarly, when fill-in responses for other text had more than 20 similar responses they were made their own category. Missing data for categorical variables (gender, race) were coded as “other.” For gender, response options with too few frequencies were collapsed into other. Therefore, the other category for gender included the following: prefer not to answer, transgender man, transgender female, non-binary, or blank. For race, all individuals who selected multiple categories were coded as being of mixed race. For participant role, we also added the category of trainer based on the filled in responses of many participants.

Participants were asked to check all pictures to indicate how rats in their care were tickled. These responses were coded for clear and consistent interpretation. Responses that only included both dorsal contact and pin were coded as Dorsal Pin Only, which is the standard, validated technique for tickling. Responses that included dorsal contact and two-handed pin were coded as Dorsal Pin Double. Responses that included dorsal contact & pin with picking the rat up and stroking it in the hands were coded as Dorsal Pin Stroke. Responses that only included a pin without dorsal contact were coded as Pin, No Dorsal. Finally, responses that only included dorsal contact or stroking without a pin were recorded as Dorsal or Stroke Only.

#### Qualitative analysis

We used thematic content analysis to determine barriers, advantages, and improvements to rat tickling [17]. Specifically, we used an inductive (bottom-up) and semantic analysis where codes were developed from the data, rather than a priori, from the explicit meanings. All coding and analyses were conducted with QSR International’s NVivo 12 qualitative data analysis software.

An iterative process was used to code the entire qualitative data set. Within the dataset, each clause was treated as the unit of analysis and each clause given a code. Each clause was coded with as many codes as it contained. For example, the clause “time and buy in from management that it is a beneficial practice” would receive codes Time and Buy-In. Buy-in was defined as a belief that rat tickling is effective and worth the time and effort it requires. Within each response, if the same code was expressed twice, the second instance would be given *Redundant* so that frequencies would accurately represent the percentage of individuals expressing a particular sentiment.

The coding manual was refined via an iterative process in which responses were read multiple times. To assess the reliability of the coding scheme, once the manual was fully established, a second rater independently coded a random 20% of the data. Inter-rater reliability was then assessed using a two-way mixed, absolute-measures intra-class correlation coefficient (ICC) [18]. The resulting ICC was in the excellent range (ICC = 0.97), which indicates that coders had a high degree of agreement and that a minimal amount of measurement error was introduced [18].

#### Quantitative analysis

Data analysis was conducted in Statistical Package for the Social Sciences (SPSS 24.0) using descriptive statistics and general linear models. Prior to testing, all assumptions of the general linear model were confirmed including independence of residuals, homogeneity of variance, normality of residuals, and multicollinearity in the data. For all summary scales, an average of individual items was calculated (excluding participants with >50% missing data in each measure).

The dependent variables for quantitative analysis were the current and planned level of rat tickling. The explanatory variables included theory of planned behavior beliefs (behavioral attitudes, subjective norms, and perceived behavioral control), familiarity with rat tickling, attitudes & behavior (Rattitude and general behaviors), enrichment (control and desire), demographic, and work factors. Additionally, to confirm the validity of the indirect measures of the theory of planned behavior, linear regression models were used between the direct and indirect factors. Significance level was p < 0.05. Results are presented as mean ± standard deviation unless otherwise noted.

## Results

### Demographics & work factors

A total of 1449 individuals started the survey, but only 924 met the inclusion criteria for this study of currently working with laboratory rats in the United States or Canada. Of those, 794 completed at least 50% of the survey and therefore were included in the analysis. Detailed demographic and work information for all participants is displayed in **Table 1**. The laboratory animal personnel were primarily white (86%) females (80%) with an average age of 40. The majority had a bachelor’s degree or higher (68%). They had worked with laboratory animals for an average of 14 years and currently worked an average of 35 hours per week with laboratory animals. In an average work week, almost half of participants (47%) spent less than 10% of their time working with rats. About two-thirds worked at a university while almost a quarter worked at a contract research organization. Finally, almost a quarter each were animal care technicians (24%), veterinary technicians (20%), or laboratory managers (20%).

**Table 1:** Demographic and work information for laboratory animal personnel (N = 794).

Categorical Data	Category	N (%)
Country	United States	557 (70%)
	Canada	237 (30%)
Gender	Female	637 (80%)
	Male	110 (19%)
	Other	8 (1%)
Race	White	680 (86%)
	Other	42 (5%)
	Asian	30 (4%)
	Black	25 (3%)
	Mixed	17 (2%)
Education	High school diploma or equivalent	18 (2%)
	Some college, no degree	69 (9%)
	Associates or technical degree	173 (22%)
	Bachelor's degree	315 (40%)
	Graduate degree	219 (28%)
Institution	University	515 (66%)
	CRO	176 (22%)
	NonProfit	37 (5%)
	Government	35 (3%)
	Other	41 (5%)
Research Type	Applied	384 (48%)
	Basic	130 (16%)
	Product	70 (9%)
	Education	65 (8%)
	Regulatory	60 (8%)
	Other	78 (10%)
Role	Animal care or laboratory technician	190 (24%)
	Veterinary technician	162 (20%)
	Manager	157 (20%)
	Veterinarian	110 (14%)
	Other	68 (9%)
	Trainer	36 (5%)
	Other research staff	34 (4%)
	Other animal care staff	25 (3%)
	Principle investigator	12 (2%)
Time with Rats	<10%	373 (47%)
	11–20%	147 (19%)
	21–30%	84 (11%)
	31–60%	103 (13%)
	61–100%	81 (10%)
**Continuous Data**	**Mean ± SD**	**Range**
Age (M +- SD)	40 ± 11 years	20–78
Years working with lab animals	14 ± 10 years	0–50
Hours per week working with lab animals	35 ± 12 hours/week	0–66

### Enrichment, attitudes, & general behaviors

**Fig 2:**
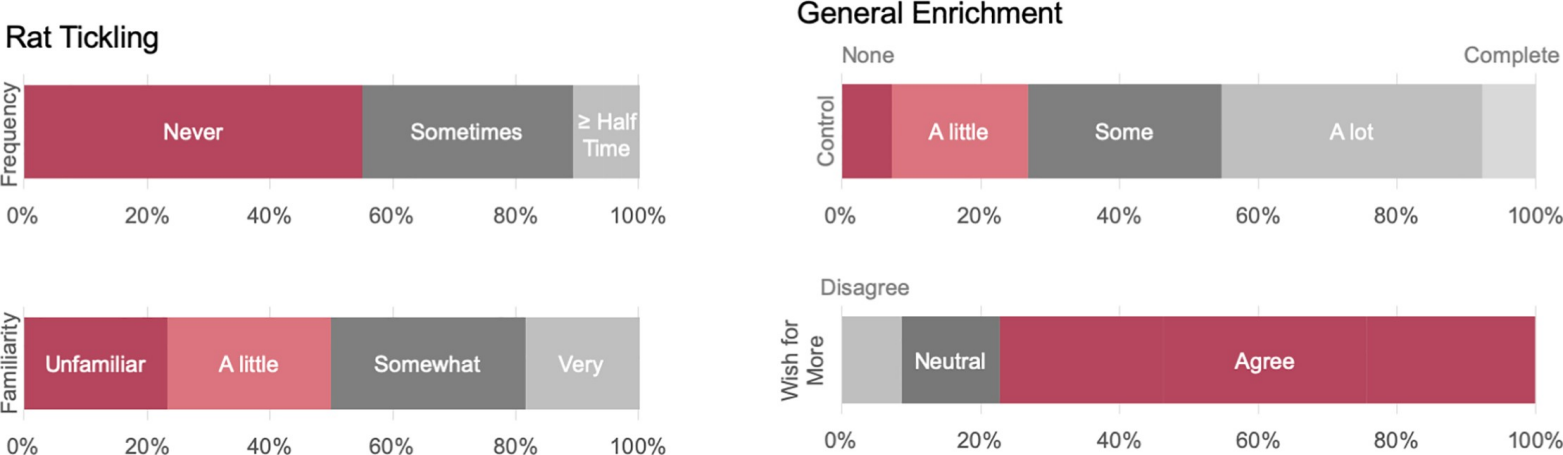
Rat tickling & general enrichment. Laboratory animal personnel’s self-reported frequency and familiarity of rat tickling, as well as general control over enrichment provision and degree to which they agree that they wish they could provide more enrichment to their laboratory animals.

Most laboratory animal personnel reported having at least a little control over enrichment (93%) and a desire to provide more enrichment (76%) (**Fig 2** and **S2 Table**). However, only 45% of participants reported having a high degree of control of enrichment (“a lot” or “complete” control). The majority of participants (>90%) have positive Rattitudes and the minority (<10%) held negative Rattitudes. Finally, the majority of participants often engaged in positive behaviors towards their laboratory animals, with a notable 41% often naming their animals.

### Rat tickling

#### Qualitative analysis of beliefs

**Fig 3:**
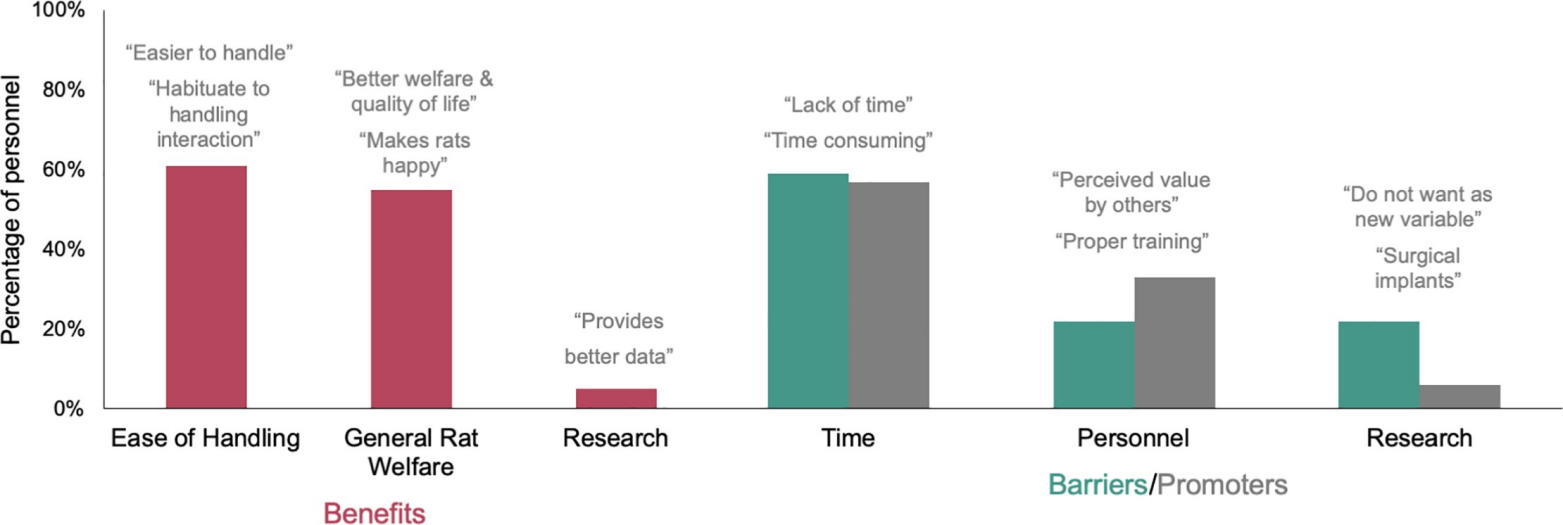
Benefits & control beliefs about rat tickling. The most common themes relating to benefits (advantages) and control beliefs (factors that are barriers that make it difficult or promoters that would make it easier) about rat tickling by 611 laboratory animal personnel currently working with rats. Graphic includes representative quotes. Sub themes and additional representative quotes are presented in **S3** and **S4 Tables**.

Participant responses to open-ended questions about rat tickling were summarized into two central categories of *Benefits* (i.e., what are the advantages to tickling rats) and *Control Beliefs* (i.e., what are the factors that make it difficult or easier to tickle rats). These central categories were further split into themes and sub-themes, described below and summarized in **Fig 3, S3 and S4 Tables**.

Participants indicated that rat tickling was beneficial primarily because it promoted *Ease of Handling* and *General Rat Welfare*, although a few participants indicated *Research* benefits or *No Benefits*. Over half of the participants indicated that rat tickling increases *Ease of Handling* and/or *General Rat Welfare*. More commonly participants specified these benefits came to both the handler and rat or, less commonly, just one species. About a tenth of these participants specifically mentioned that rat tickling promotes a bond. About a quarter of the overall participants specifically indicated that rat tickling reduces rat stress, anxiety, or fear. Additionally, 21% of participants indicated that rat tickling was a form of enrichment, with some specifying it was particularly good for socialization. Conversely, less than 10% of participants indicated that rat tickling was beneficial for *Research* or *No Benefits*. Some participants indicated rat tickling can improve research data or outcomes. A few participants did not think it was beneficial at all for a particular study or compared to other enrichment techniques–alternatively some participants simply did not know enough to indicate benefits.

Almost 60% of participants indicated that *Time* was a key factor controlling rat tickling implementation. Most indicated that the time required made rat tickling difficult or that more time would make it easier. Additionally, some participants specifically mentioned time related factors such as limits imposed by *Staffing*, *Number of Rats*, or the *Consistency* needed for the technique. Despite the direct relationship between staff time and money to pay those staff, few participants mentioned *Money* as a limiting factor.

The second most common control beliefs were *Personnel* and *Research*. Within the theme of *Personnel* participants stated that a lack of *Buy-in* and *Education* of staff or (rarely) even *A Fear of Rats* may make rat tickling difficult, but that promoting *Buy-in* and *Education* may make it easier. Within the sub-theme of *Buy-In*, some personnel specifically stated that they thought implementing the technique was *Not My Problem* as it was not in their role or that *Official Approval* such as via IACUC (institutional animal care and use committee) or Principle Investigators would be beneficial to promote rat tickling. Within the sub-theme of *Education*, participants indicated general *Awareness* and lack of *Training* make rat tickling provision difficult and that increasing awareness and training opportunities would help. Within the theme of *Research–*which was more than three times more likely to be cited as a barrier than promoter–participants indicated concerns with introducing a *New Variable* to studies, that specific study-related *Rat Factors* or *Research Protocols*, or even just a *Short Study* may make implementation of rat tickling more difficult.

Participants less commonly mentioned control beliefs relating to *Rats*, *Safety*, *or Facility Factors*, and, of course, a few participants either indicated there being *No Barriers* or *Nothing Easier*. Within the theme of *Rats*, a variety of specific problems such as *Age* (older rats being less receptive), *Aggression* levels, *Individual Differences*, *Single-Housing*, *and Breeding Status*. Within the theme of *Safety*, participants mentioned concerns about maintaining *Biosecurity* or rat tickling resulting in *Harm to Personnel* either through bites or zoonotic diseases. Within the theme of *Facility Factors*, individuals indicated factors related to wanting more space in the rooms or housing rats in *Larger Cage Sizes*.

#### Quantitative analysis

Overall, participating laboratory animal personnel reported being fairly unfamiliar with rat tickling (50%) and the majority never tickle their rats (55%; **Fig 2** and **S2 Table**). For those reporting using it, most do not report using standard, validated technique of a dorsal contact and single-handed pin (55%). Conversely, 21% of personnel indicated that rats were only given a dorsal contact in the cage or stroked in the hand, 17% indicated that rats were only pinned with one or two hands but did not receive dorsal contact, and 11% indicated that rats were tickled with a dorsal contact and either single or double-handed pin and also stroked. The rest of the participants indicated some combination of techniques, that they were unsure, or that the technique was not pictured.

**Fig 4:**
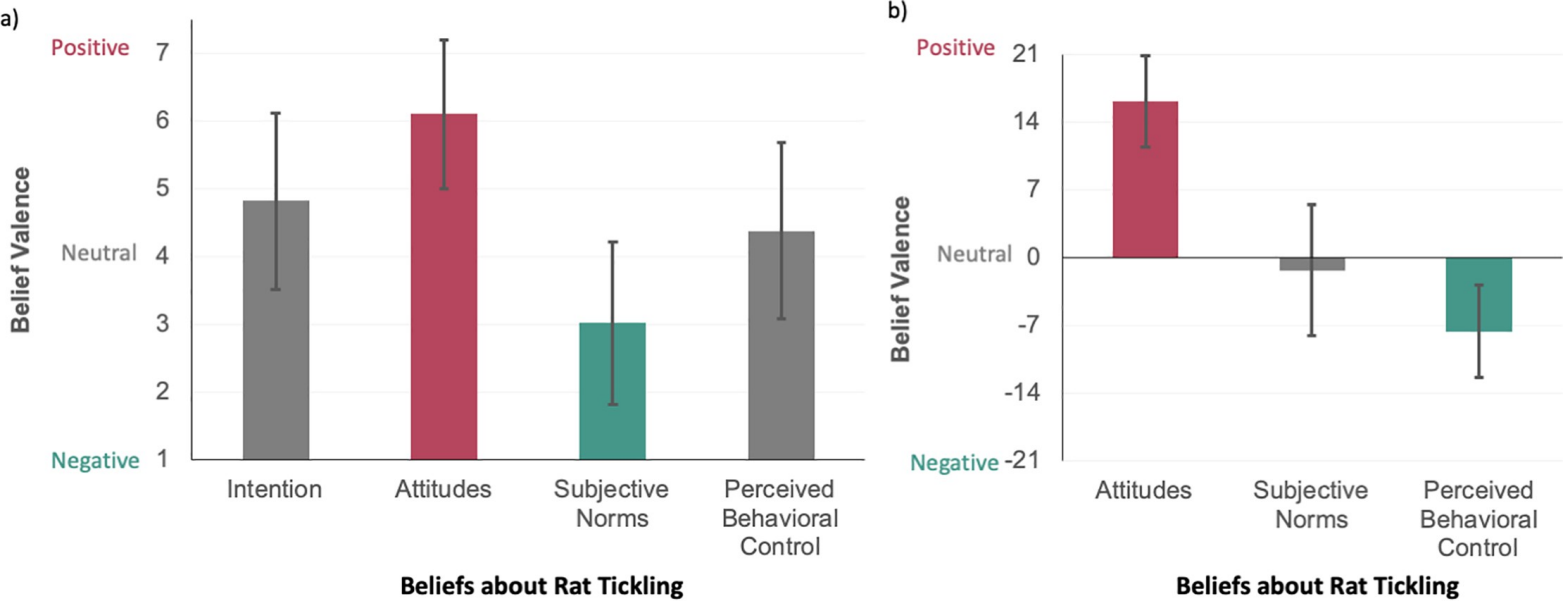
Beliefs about rat tickling. Laboratory animal personnel (N = 656) self-reported intention to provide and beliefs about rat tickling (M ± SD). All scales were developed from Theory of Planned Behavior protocols including beliefs about the consequences (behavioral attitudes), social and professional pressures (subjective norms), and control over (perceived behavioral control) providing rat tickling (a. direct, b. indirect).

For quantitative analysis, 656 and 591 participants were included for current and planned implementation of rat tickling, respectively. The former completed at least 50% of each scale in the quantitative theory of planned behavior section and the latter also reported their current level of rat tickling. These participants reported a very slightly positive *intention* to tickle rats in the next year (**Fig 4 and S5 Table)**. From both direct and indirect measurements, participants had overall positive *attitudes* (e.g., they think rat tickling is good and are in favor of providing it), were relatively neutral to negative *subjective norms* (e.g., they do not feel social pressure to provide rat tickling), and neutral to negative *perceived behavioral control* (e.g., they are not confident they can provide rat tickling and there are barriers; **Fig 4, S5 and S6 Tables**). In terms of measurement reliability, our direct measures had acceptable reliability within scales (**S5 Table**; Cronbach’s alpha >0.7 except for perceived behavioral control alpha = 0.66) and our indirect measurements were significantly associated with the direct measures (**S6 Table;** significant standardized regression coefficients, p < 0.01).

**Fig 5:**
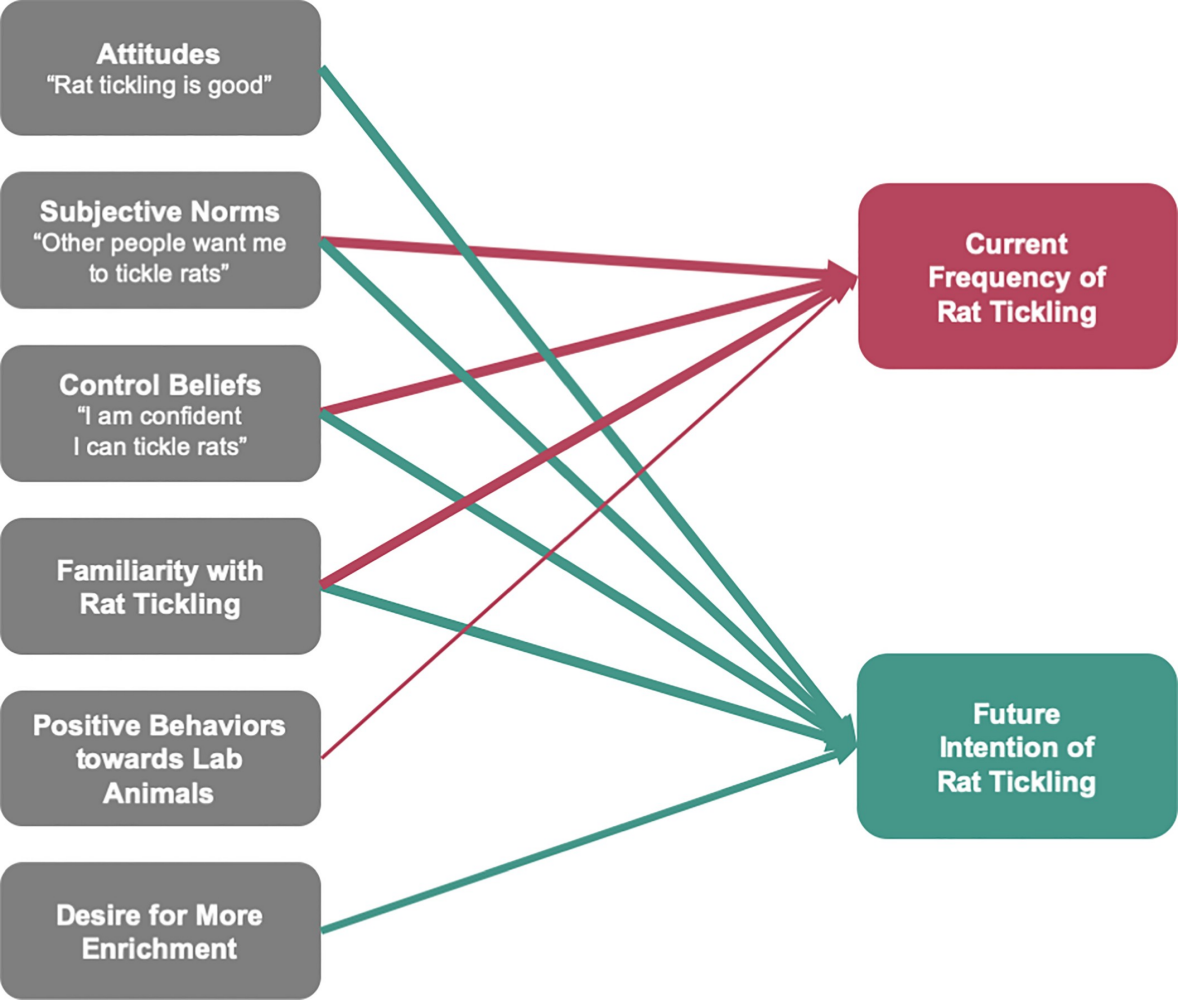
Significant associations between moderator factors and rat tickling implementation. This figure shows significant associations from self-report data from laboratory animal personnel about potential moderating factors and both current (top, N = 591) and future (bottom, N = 656) rat tickling. Lines only connect between significant associations. The thickness of the lines indicates the significance of association (thick = p < 0.0001, medium = p < 0.001, thin = p < 0.05). Non-significant moderators included positive and negative Rattitude and control over enrichment. Models were run controlling for age, years working, hours of work per week, % of time working with rats, gender, role, institution, research type, race, and highest education (none which were significant). Numerical data is reported in S7 Table.

In this study, current and planned rat tickling was associated with several factors (**Fig 5 and S7 Table**). Both current and planned rat tickling were positively associated with subjective norms, control beliefs, and familiarity. Current and planned rat tickling were also positively associated with more positive attitudes towards laboratory animals in general or both more positive attitudes towards rat tickling and a higher desire to implement more enrichment in general, respectively. Additionally, working in Canada was positively associated with current rat tickling implementation (β = 0.0249, p = 0.002).

## Discussion

To our knowledge, this study is the first to quantify current rat tickling prevalence and potential factors potentially influencing its implementation in the laboratory research environment. We successfully surveyed 794 laboratory animal personnel working in a variety of roles, institutions, and research types. Our results indicate that laboratory animal personnel report relative unfamiliarity with and infrequent implementation of rat tickling. When implementation of rat tickling is reported, the technique used is often indicate not the standard, validated one. Overall, our analyses indicate that although laboratory animal personnel generally believe rat tickling is beneficial, they do not feel confident in their ability to provide it due to lack of time and education. They do not feel social or professional pressure to provide this enrichment. On the contrary, there is indication that, they may even feel pressure not to provide it by other personnel or research staff (rather than neutral ambivalence). However, there is a positive association between more positive beliefs about, as well as familiarity with, rat tickling its implementation. There are also positive associations between implementation of rat tickling and general positive behaviors towards lab animals and a general desire to provide more enrichment.

### Current rat tickling implementation

In March of 2018, laboratory animal personnel in this population reported mostly no or low familiarity with and implementation of rat tickling. Although rat tickling originated in 1999 and has over 32 publications [7], the technique was not published as a habituation technique until 2008 [19], with its evidence base synthesized until 2017 [7], and with a peer-reviewed video standard operating procedure until May 2018 (after this survey was administered) [20]. It is possible that peer reviewed journal articles are not the almost effective form of communication for busy laboratory animal personnel.

Furthermore, even when personnel did report implementing rat tickling, many indicated using techniques that do not mimic aspects of rat rough-and-tumble play with a dorsal contact and pin [20]. For example, 21% of participants indicated that their rats are “tickled” with only a dorsal contact, similar to stroking or petting. Unfortunately, stroking or light touch can be aversive to naïve rats, eliciting vocalizations indicative of negative affect [5] and fewer positive outcomes [7]. Using non-validated techniques such as these may result in fewer positive results and even negative results (e.g., defensive posturing). In turn, this could contribute to less positive attitudes towards and implementation of rat tickling.

### Beliefs & associations of rat tickling

Laboratory animal personnel overall had positive behavioral attitudes about rat tickling–that is, they generally thought that rat tickling is good and beneficial. Furthermore, participants who had higher positive behavioral attitudes about the technique were also more likely to plan to provide rat tickling in the next year. In particular, rat tickling was cited to be beneficial for rat welfare and handling (but rarely research aims) in both qualitative and quantitative data. This may be a result of publications and video evidence demonstrating the handling and welfare benefits of rat tickling [7], while there are no publications showing improved research factors. Furthermore, handling and welfare benefits may be more salient to the laboratory animal personnel surveyed (only 2% of participants were Principle Investigators), while research factors may be more often a constraint. Regardless, it may be beneficial to develop research and case studies showing the feasibility and benefits of rat tickling for scientific research.

Overall, personnel had very low subjective norms about rat tickling–that is, they generally felt little to no social or professional pressure to provide this technique. However, participants who held higher subjective norms about rat tickling were more likely to indicate higher levels of current and planned rat tickling. Specifically, personnel may find the opinions–and social pressure–of accreditation staff, laboratory animal veterinarians, study leads, and principle investigators particularly important. In fact, some personnel indicated they felt study leads in particular should be responsible for initiating rat tickling implementation. Related to the current lack of professional pressure to provide rat tickling, some personnel cite its name “rat tickling” as a barrier to implementation. Our research team also promotes the term “heterospecific play” which is more commonly used in neuroscience publications [21].

Perceived behavioral control over providing rat tickling was overall reported to be neutral to negative–that is, that personnel do not feel in control of providing rat tickling. However, personnel who reported more positive control beliefs were also more likely to indicate higher levels of current and planned rat tickling. That is, individuals who felt confident that they *could* implement the technique were more likely to tickle rats. Specifically, personnel believe that having enough time, official approval, and sufficient training are very important. A lack of time was by far the most commonly cited barrier for rat tickling. This is unsurprising considering typical protocols recommend 2 min of tickling per rat for 5 days (10 min total per rat) [7]. As our research team predicted this barrier, we completed a study (published 6 months *after* collecting this data) that reduces the time requirement down to 15 s for 3 days per rat (45 s total per rat) [8]. While this recommendation still requires additional time, it should be significantly more manageable. Additionally, 3 days of tickling can easily fit within an institution’s mandated, post-shipment, acclimation time, and prior to the start of the study.

In addition to beliefs about rat tickling, we also found positive associations between familiarity with rat tickling, behaviors towards laboratory animals (but not Rattitude), general enrichment desires, and rat tickling implementation. Unsurprisingly, a greater familiarity with the practice of rat tickling was a strong predictor of both current and future intention of rat tickling. More positive general behaviors (e.g. talking to or naming laboratory animals) predicted current rat tickling implementation. Laboratory animal personnel who perform these behaviors at higher levels may be highly motivated to seek out and implement enrichments such as tickling. These results mirror findings that farm animal stockpeople with more positive general behaviors towards their farm animals also have more positive human-animal interactions with them [11]. Conversely, attitudes towards rats in general (e.g. beliefs that rats are smart, curious) was not associated with rat tickling implementation, which may be a result of a ceiling effect in that this sample had overwhelming strong positive and weak negative Rattitude (i.e. attitude towards rats). Finally, unsurprisingly, individuals with a stronger desire to provide more enrichment had a higher intention to implement of rat tickling in the future. Overall though, our study did not find any associations between work factors, demographic variables, and rat tickling implementation. This may indicate that it is feasible to implement rat tickling regardless of institution, research, or personnel.

Interestingly, in response to open-ended questions, some personnel indicated that using rat tickling to form a bond between handler and rat would be beneficial. However, creation of this bond was never cited as a barrier or disadvantage to rat tickling. Although research encourages bonds between laboratory animals and personnel [22], anecdotally we have heard that personnel may be hesitant to tickle rats because of fear that establishing a bond could make aversive procedures more difficult, and, in turn, increase compassion fatigue. In actuality, this does not seem to be a concern. Additionally, using further data collected during this survey (LaFollette et al., In Preparation), no association was found between frequency of rat tickling and compassion fatigue (burnout or secondary traumatic stress).

### More, better training is needed

Taken together, our results indicated there is a need for greater tickling education to promote implementation. Previous studies show that researchers can change personnel behaviors by holding trainings that directly target both behavior and beliefs [13]. Educating laboratory animal personnel should not rely on peer-reviewed publication but include targeted training using appropriate educational theories to maximize behavior change. Based on this survey, training may include focus areas such as teaching participants proper technique while increasing their confidence, addressing common concerns about implementation, emphasizing its benefits to improve attitudes, and even emphasizing that rat tickling may become a social norm. Any efforts to increase perceived behavioral control (e.g. hands-on instruction in the technique or reducing time required) are likely to be particularly well received. Educating accreditation staff, veterinarians, study leads, and individuals particularly interested in enrichment may be particularly effective to increase social norms and professional pressure. Furthermore, it would be beneficial to show more evidence of rat tickling being used successfully in typical research studies, hopefully to the benefit of research data.

### Limitations

There were several limitations to this project. First, since this study was cross-sectional it is impossible to determine the causation of any associations that we found. For example, perhaps the ability to currently implement rat tickling causes higher positive control beliefs, rather than more control beliefs causing higher current implementation of rat tickling. Future studies would benefit from randomly assigning laboratory animal personnel to educational workshops designed to change personnel beliefs to determine the causality of the association. However, this study provides insight into what those educational workshops could contain.

Second, since this survey only involved self-report data from laboratory animal personnel, there is the potential for subjective biases to occur. Our team did not directly measure the level of rat tickling, personnel behavior, or animal welfare. Therefore, it is possible that the personnel may have over or underestimated their current level or future ability to provide rat tickling. Future studies could implement a diary tracking method or allow for follow-up to see if rat tickling is indeed implemented with more positive attitudes and higher intentions. However, this study provides valuable broad, exploratory insight into the perspectives of laboratory animal personnel and how those beliefs may impact enrichment implementation.

Finally, as this was a voluntary, survey-based convenience sample study, we are unsure if our sample is representative of the population or if participants were affected by sampling bias. One factor to consider is that we only translated the survey into French and not Spanish. It is unknown whether this low percentage of Hispanic and Latino participants is truly representative of the laboratory animal personnel field. If not, these participants could have characteristically different attitudes towards rat tickling. Regardless, the very large sample size obtained suggests that we have accurately characterized attitudes towards rat tickling.

## Conclusions

In conclusion, as of May 2018, rat tickling appears to be relatively rarely implemented by laboratory animal personnel. Although laboratory animal personnel may believe rat tickling is beneficial for rat handling and welfare, they also believe there are key barriers to its implementation in the form of time, personnel, and research. Furthermore, there are statistical associations between higher current and planned rat tickling implementation and factors such as higher positive beliefs (attitudes, subjective norms, and control beliefs), familiarity with tickling, general positive behaviors to lab animals, and a desire to implement more enrichment. Laboratory animal personnel beliefs seem to be a key to promoting the widespread implementation of beneficial enrichment techniques. Overall, our results suggest that further research on reducing the time required to tickle rats, increasing personnel buy-in and education (therefore improving attitudes, subjective norms, control beliefs, and familiarity), and showing the benefit of rat tickling for research could help increase its prevalence and improve laboratory rat welfare.

## Supporting information

S1 Table Rat tickling survey questionnaire. The question scale, question text, response options, and coded response values of the survey given to laboratory animal personnel.
(XLSX)

S2 Table Laboratory animal personnel enrichment, attitudes, & rat tickling practices. Laboratory animal personnel self-reported enrichment factors (control over enrichment and desire for an increased level of enrichment in their laboratory animals), attitudes towards laboratory rats, attitudes towards all animals in their care, and familiarity with as well as current level of rat tickling. N indicates the number of participants who completed the question and met inclusion criteria for the survey. Range indicates the lowest to highest value selected by participants, which in the case of average summary statistics was not always a whole number.
(XLSX)

S3 Table Benefits to rat tickling. The themes relating to advantages to rat tickling as described by 611 laboratory animal personnel (number and percent) currently working with rats. Main themes are indicated in bold, sub-themes in normal text, and themes within sub-themes in italics.
(XLSX)

S4 Table Control beliefs about rat tickling. The themes relating to perceived control beliefs to rat tickling as described by laboratory animal personnel (number and percent) currently working with rats. Responses to two questions asking participants to indicate any factors that either make it more difficult (Barriers) or easier (Promotors) to provide rat tickling. Main themes are indicated in bold, sub-themes in normal text, and sub sub-themes in italics.
(XLSX)

S5 Table Direct beliefs about rat tickling and reliability of scales. Laboratory animal personnel (N = 656) self-reported intention to provide and beliefs about rat tickling. All scales were developed from Theory of Planned Behavior protocols including beliefs about the consequences (behavioral attitudes), social and professional pressures (subjective norms), and control over (perceived behavioral control) providing rat tickling. All items measuring each construct are presented with the summary score (in bold) and individual item mean and standard deviation and Cronbach’s alpha between individual items. All scales except behavioral attitudes range from 1 to 7 where 1 = strongly disagree, 4 = neutral, and 7 = strongly agree. The behavioral attitudes measure ranges from the end points indicated in the table. ^Reverse coded *Deleted after reliability test.
(XLSX)

S6 Table Indirect beliefs about rat tickling and their associations to direct measurements. Laboratory animal personnel (N = 656) self-reported beliefs about rat tickling including beliefs about the consequences (behavioral attitudes), social and professional pressures (subjective norms); and control over (perceived behavioral control) providing rat tickling to laboratory rats. All items measuring each construct are presented with the summary (in bold) and individual item mean and standard deviation. Additionally, the association (standardized regression coefficients, β) between each indirectly and directly measured summary scale is presented. The possible range of all scores is from -21 to +21 since they are calculated by multiplying one scale running 1 to 7 and another scale from -3 to +3 (specific scales are indicated in the table on sheet 2). **p < 0.01 ***p<0.001.
(XLSX)

S7 Table Descriptive statistics and moderator analysis of factors related to rat tickling implementation. The mean (M), standard deviation (SD) and associations (standardized regression coefficients, β) from self-report data from laboratory animal personnel about their rat tickling beliefs (using the theory of planned behavior) and familiarity, attitudes towards rats & laboratory animals, enrichment control & desire, and demographic & work factors. Dependent variables were the current level & future intention of providing rat tickling. Models were run controlling for gender, role, institution, research type, race, and highest education (none which were significant). Bold indicates a significant effect. *p < 0.05, **p < 0.001, ***p < 0.0001.
(XLSX)
